# Concurrent *IDH1* and *SMARCB1* Mutations in Pediatric Medulloblastoma: A Case Report

**DOI:** 10.3389/fneur.2018.00398

**Published:** 2018-06-19

**Authors:** Moatasem El-Ayadi, Kristof Egervari, Doron Merkler, Thomas A. McKee, Fabienne Gumy-Pause, Damian Stichel, David Capper, Torsten Pietsch, Marc Ansari, André O. von Bueren

**Affiliations:** ^1^Division of Pediatric Hematology and Oncology, Department of Pediatrics and Adolescent Medicine, Geneva University Hospitals (HUG), Geneva, Switzerland; ^2^CANSEARCH Research Laboratory, Department of Pediatrics, Faculty of Medicine, University of Geneva, Geneva, Switzerland; ^3^Department of Pediatric Oncology, National Cancer Institute, Cairo University, Cairo, Egypt; ^4^Department of Pediatric Oncology, Children Cancer Hospital of Egypt, Cairo, Egypt; ^5^Department of Pathology and Immunology, Faculty of Medicine, University of Geneva, Geneva, Switzerland; ^6^Division of Clinical Pathology, Geneva University Hospitals (HUG), Geneva, Switzerland; ^7^Clinical Cooperation Unit Neuropathology, German Cancer Consortium, German Cancer Research Center (DKFZ), Heidelberg, Germany; ^8^Department of Neuropathology, Charité — Universitätsmedizin Berlin, Corporate Member of Freie Universität Berlin, Humboldt-Universität zu Berlin, and Berlin Institute of Health, Berlin, Germany; ^9^Partner Site Berlin, German Cancer Consortium (DKTK), German Cancer Research Center (DKFZ), Heidelberg, Germany; ^10^Institute of Neuropathology, Brain Tumor Reference Center, Deutsche Gesellschaft für Neuropathologie und Neuroanatomie, University of Bonn Medical Center, Bonn, Germany

**Keywords:** medulloblastoma, IDH-1, SMARCB1, SHH activation, pediatric, mutation

## Abstract

Isocitrate Dehydrogenase-1 (*IDH1)* is a driver gene in several cancers including brain tumors such as low-grade and high-grade gliomas. Mutations of *SMARCB1* were described in atypical teratoid rhabdoid tumors and to date have not been associated with the pathogenesis of medulloblastoma. We report concurrent *IDH1* and *SMARCB1* mutations in a medulloblastoma patient. We searched the catalog of somatic mutations in cancer (COSMIC) database and other mutation databases and -to our knowledge- this is the first reported case of medulloblastoma harboring both mutations together. Our patient is a 13-year-old male presenting with headache and vomiting at diagnosis. MRI revealed left cerebellar expansive lesion with no evidence of metastasis. A histopathological diagnosis of desmoplastic/nodular medulloblastoma was made after complete resection of the tumor. Immunophenotypic characterization and methylation profiling suggested a medulloblastoma with SHH activation. Next generation sequencing of a panel of 400 genes revealed heterozygous somatic *IDH1*(p.R132C), *SMARCB1*(p.R201Q), and *CDH11*(p.L625T) mutations. The patient was treated according to the HIT-SIOP PNET 4 protocol. He is in complete remission more than 2 years after diagnosis. In conclusion, increasing use of high throughput sequencing will certainly increase the frequency with which rare mutations or mutation combinations are identified. The exact frequency of this mutation combination and whether it has any particular therapeutic implications or prognostic relevance requires further investigation.

## Background

Medulloblastoma (MB) is a highly malignant embryonal tumor of the cerebellum and represents the most frequent malignant brain tumor of childhood (1). Molecular subgroups of MB had been described in 2012, including the wingless (WNT) group of tumors with WNT signaling pathway activation and the sonic hedgehog (SHH) group with aberrant activation of SHH signaling pathway as well as groups 3 and 4 tumors (2). This was refined by the recent revised WHO classification for tumors of the CNS 2016 in which four genetically defined MB entities have been defined that differ in cell of origin, genetic alterations and pathway activation as well as in histopathological hallmarks and clinical behavior (1). These are MB-WNT, MB-SHH/TP53 wild type, MB-SHH/TP53 mutant and non-WNT/non-SHH MB (with its variants Grp3 and Grp4, not considered as entities but overlapping variants). Several recurrent mutations were recognized in these 4 MB entities including mutations of *CTNNB1, PTCH1, SUFU, SMO, TP53, MLL2, SMARCA4, DDX3X, CTDNEP1, KDM6A, TBR1*, and other genes (3, 4).

*IDH1* is a gene located at 2q33.3 that encodes cytoplasmic isocitrate dehydrogenase, which is involved in the control of cellular oxidative damage. *IDH1* is detected as a mutational cancer driver in several cancer types including diffuse low-grade and high-grade gliomas but until recently not in MB (5–8). *SMARCB1* (also known as *hSNF5*/*INI1)* is a tumor suppressor gene located at 22q11.23. Inactivating mutations of *SMARCB1* were previously described as a hallmark event in atypical teratoid rhabdoid tumors (ATRT) (9) and such mutations were ruled out from being involved in the pathogenesis of MB (10).

Here, we report concurrent *IDH1* and *SMARCB1* mutations -among other mutations- in a case of a pediatric desmoplatic/nodular MB with SHH activation. We searched for reported *IDH1* and *SMARCB1* mutations in MB within the catalog of somatic mutations in cancer (COSMIC) and other mutation databases. To our knowledge, this is the first reported case of MB that harbor both mutations together; each of these mutations is very rarely reported in MB.

## Case report

**Figure 1:**
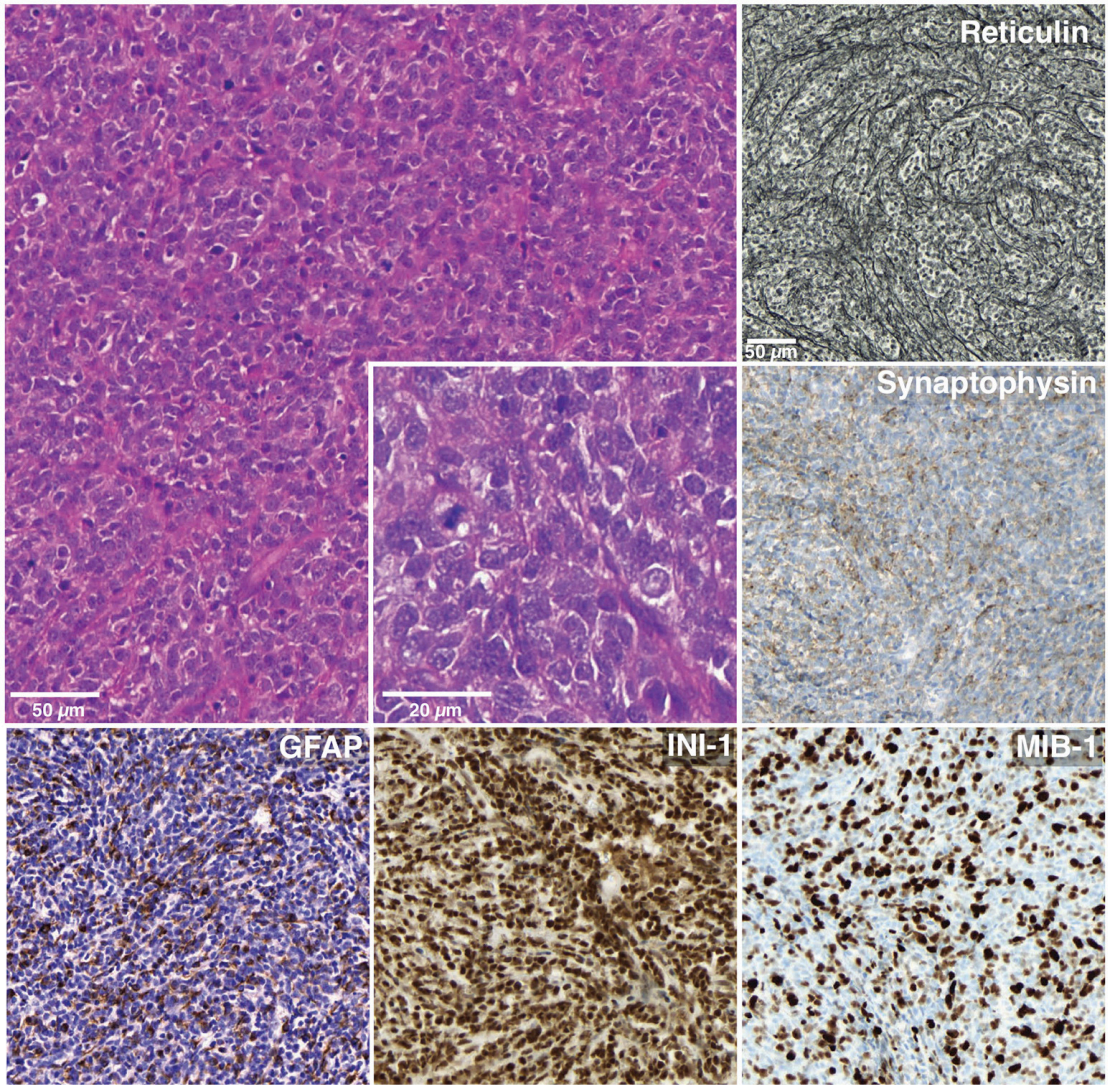
Histology and immunohistochemistry. Hematoxylin-eosin stained image from a representative area of the tumor shows a small to medium sized primitive cellular population. A desmoplastic micronodular architecture is revealed by reticulin staining. The tumor cells are positive with synaptophysin and weakly with GFAP. INI-1 expression is preserved. The proliferative index (MIB-1) reaches 70%.

**Figure 2:**
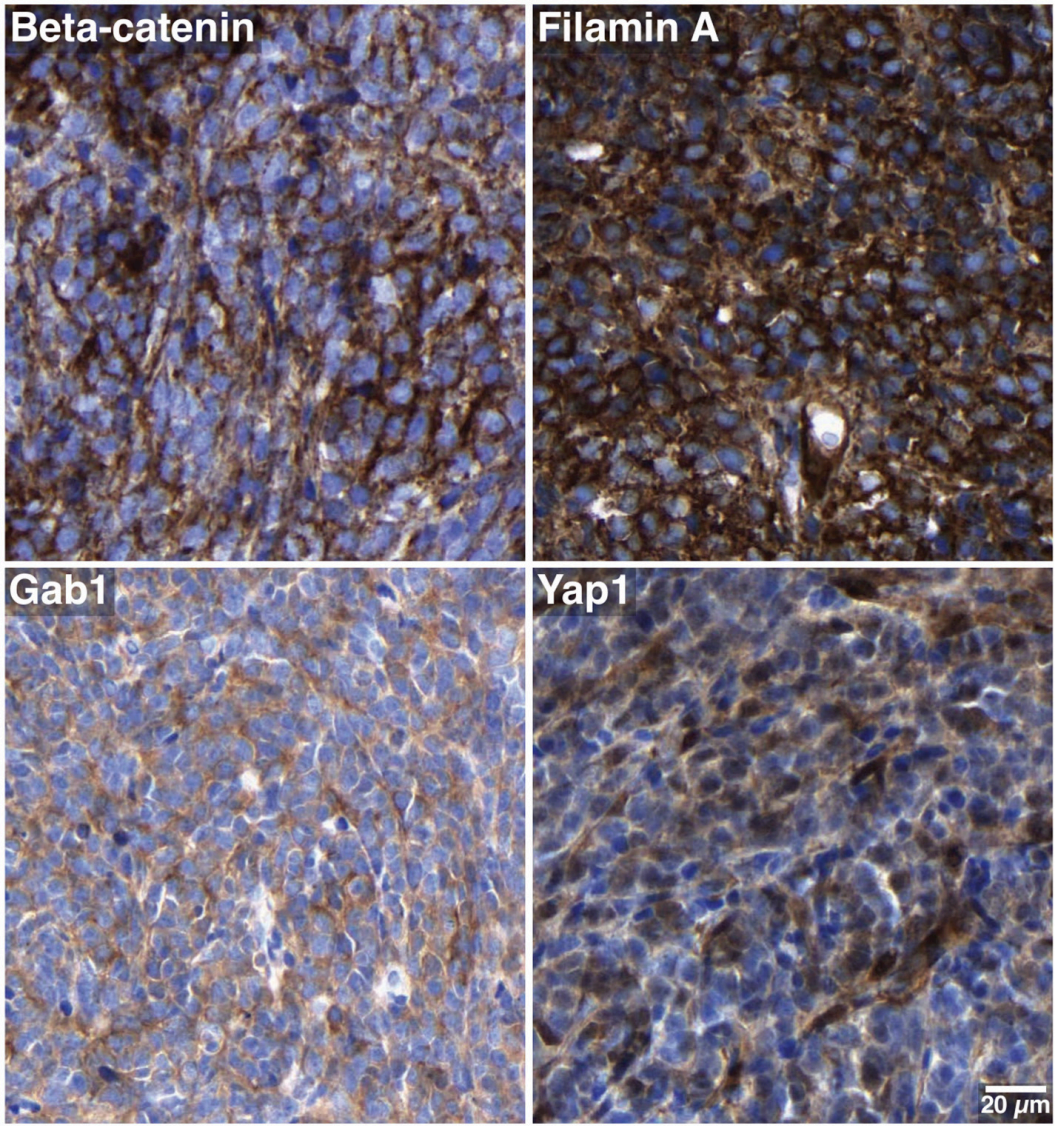
Subgroup-specific immunohistochemical markers. The tumor cells show only cytoplasmic but no nuclear beta-catenin positivity, and are stained with antibodies against filamin A, Gab1 and Yap1. These characteristics are consistent with SHH MB.

**Figure 3:**
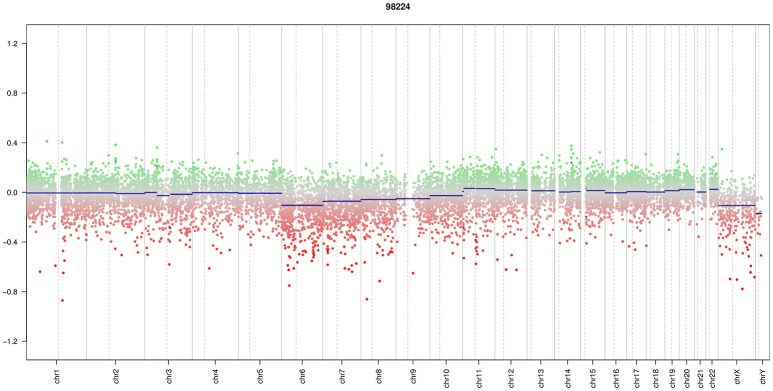
Copy number profile. Array data showed a low density copy number profile which indicates an almost flat genome without relevant chromosomal aberration.

**Figure 4:**
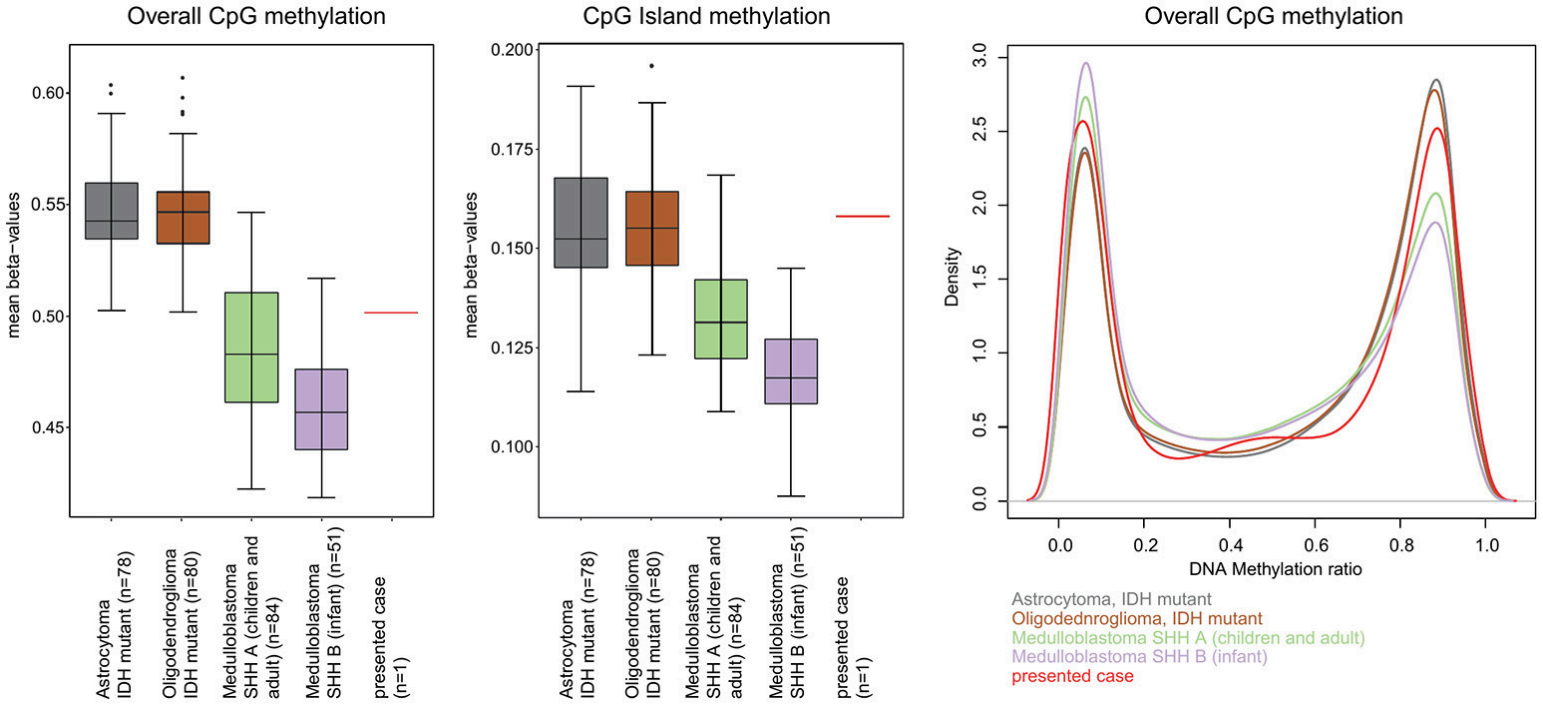
Assessment of overall CpG methylation and CpG island methylation levels. **(Left)** The distribution of mean beta-values for all CpG-probes of the EPIC methylation array is displayed as box-plots for IDH mutant astrocytoma, IDH mutant oligodendroglioma, medulloblastoma SHH A (children and adult), medulloblastoma SHH B (infant) and the presented case. IDH mutant astrocytoma and oligodendroglioma are given for comparison because of the known CpG Island Methylator Phenotype (CIMP) associated with IDH mutations in these tumors. The overall CpG methylation of our presented cases was not higher than that in other medulloblastoma groups and lower than that in the CIMP tumors. **(Middle)** When just the CpG island associated CpG sites were analyzed, the mean beta value of our case was clearly higher than that of other medulloblastoma of the same age group and was well in the range of the other CIMP tumors indicating some degree of CpG island hypermethylation. **(Right)** Density plot analysis of the distribution of beta-values (“methylation ratio”) of all CpG probes also indicated a moderate relative hypermethylation.

A 13-year-old boy presented with a history of headache, nausea and vomiting with an acute onset 2 weeks earlier. Magnetic Resonance Imaging (MRI) of the brain and spinal cord revealed left cerebellar expansive lesion with no evidence of metastasis. Cerebrospinal fluid (CSF) examination revealed no evidence of dissemination. He underwent complete surgical resection as confirmed by postoperative imaging. Histopathological analysis including reticulin staining revealed a desmoplastic/nodular MB (confirmed by a central review by T.P.) as shown in Figure 1. Diffuse severe cytological anaplasia was not present. Complementary immunophenotypic characterization as described (11, 12) suggested a MB with SHH activation, *TP53* wild-type (Figure 2). Of note, nuclear INI-1 staining was preserved (Figure 1) while P53 immunostaining showed nuclear positivity only in a small proportion of the tumor cells (data not shown). There was no evidence of *MYCN* or *MYCC* amplification by fluorescence *in-situ* hybridization (FISH). Next generation sequencing (NGS) over a panel of 50 genes (Ion AmpliSeq™ Cancer Hotspot Panel v2)^1^ revealed *IDH1* R132C mutation in 46% of cells. NGS was repeated over a panel of 400 genes (Ion AmpliSeq™ Comprehensive Cancer Panel)^1^ and it revealed *IDH1* R132C mutation in 24% of cells as well as *SMARCB1*-R201G in 30% of cells and *CDH11*-L625T in 26% of cells (Table A1). The panel was tested on both tumor and normal tissue to confirm the somatic nature of the mutations. Of note, mutations in *SMO, PTCH1, SUFU* and *TP53* were not detected. Infinium Methylation EPIC BeadChip (850k) array revealed highest resemblance to the methylation class MB, subclass SHH A (children and adult). However, the calibrated score was 0.44 so that a clear subgroup assignment could not be done (13). Calculation of a low density copy number profile from the array data indicated a flat genome without relevant chromosomal aberration (Figure 3). Deletions of chromosome 9q (PTCH1) were absent. Assessment of overall CpG methylation and CpG island methylation levels of the tumor showed relative CpG hypermethylation compared to other reference classes of MB (13) (Figure 4). There was no family history indicating a tumor predisposition syndrome.

The patient started treatment according to the HIT-SIOP PNET 4 protocol on the standard radiotherapy (RT) arm (14) where he received cranio-spinal RT (23.4 Gy) with boost to the posterior fossa up to 55.8 Gy in 30 fractions over 42 days. Adjuvant chemotherapy started 6 weeks after the end of RT and it includes eight cycles of maintenance chemotherapy (14). Treatment was well tolerated with no major life-threatening adverse events or dose limiting toxicities. To date, the patient is in complete remission more than 2 years since diagnosis.

Several mutation databases were searched, namely; the catalog of somatic mutations in cancer (COSMIC) database (15), the cBioPortal for cancer genomics database (16), the integrative onco genomics (IntOGen) database for mutational cancer drivers (17), the cancer genetics web database (18) as well as the medulloblastoma advanced genomics international consortium (MAGIC) project (19). We checked for reported *IDH1* and *SMARCB1* mutations in MB samples.

The COSMIC database reports on *IDH1* mutation in only 5 tumor samples from 4 patients with MB; three samples from two adult cases (20, 21) and two samples from two pediatric patients [(8), ICGC PedBrain Tumor Project; unpublished data]. Searching other databases didn't reveal any additional reported cases.

As for the *SMARCB1* mutation, the COSMIC database reports only on 9 mutated samples of MB from four different studies (10, 22–24). Similarly, we couldn't find any additional reported cases in other mutation databases. The specific mutation (*SMARCB1*-R201G) was only reported in a single case of ovarian cancer, and wasn't previously reported in any central nervous system (CNS) tumor.

There was no reported case of a MB harboring both mutations of *IDH1* and *SMARCB1* in any of the above mentioned databases.

## Discussion

Our 13-year-old patient had a desmoplastic/nodular MB with SHH activation and without TP53 alteration. Around 25% of MB show SHH activation, most of which show desmoplastic/nodular histology, though classic or large-cell/anaplastic (LCA) histologies are seen too (25). SHH-MB frequently arise from external granule cell progenitors in a cerebellar hemisphere (26, 27). Childhood SHH-MB frequently harbor *TP53* mutations (48%), *PTCH1* mutations (36%) or *SMO* mutations. *TP53* mutations are commonly germline (80%) as part of Li-Fraumeni syndrome, and they can carry *MYCN* (42%) and/or *GLI2* amplifications (28). None of these mutations were found in our case, and *MYCN* amplification was ruled out by FISH. In contrast to SHH -TP53 mutant MB which represent the most aggressive MB entity, SHH-TP53 wild-type MB usually show a more favorable clinical course. This might -in part- explain the apparently good outcome of our patient who is still alive and in complete remission for more than 2 years since diagnosis. It remains less evident which genetic event cause the SHH activation in this case.

Several studies investigated *IDH1* mutations among different types of cancers (6, 29–32). Hayden et al. analyzed the combined results of five different studies with more than 2,650 tumor samples of different cancer types including 1,603 samples of brain tumors (33). They found 37% of CNS tumors with *IDH1* mutations—all involving codon 132—with highest frequency of mutations found in diffuse astrocytic or oligodendroglial tumors (81%) (33). Notably, no *IDH1* mutation was found among 113 cases of MB (33). Similarly, the International Cancer Genome Consortium (ICGC) PedBrain Tumor Project examined samples of 125 pediatric MB patients using whole genome sequencing (WGS) and whole-exome sequencing (WES) (24). None of the examined cases showed *IDH1* mutations. However, another large sequencing study on 92 MB samples reported a single case with *IDH1* R132C mutation in a 13 years old boy with WNT-MB (8). An adult study about *CIC* and *FUBP1* mutations in primary brain tumors reported another case of MB with an *IDH1* R132H mutation (21). In 2015, Snuderl et al. reported an adult SHH-MB case with *IDH1* R132S mutation using deep sequencing. They also identified another case with an *IDH1* R132H mutation using immunohistochemistry. Interestingly, a recent analysis of 491 sequenced MB samples identified six *IDH1-*R132C mutations with five of them in SHH activated MB (4).

Somatic mutation of the *IDH1* gene was found to establish CpG island methylator phenotype (CIMP) in *IDH*-mutant astrocytomas and oligodendrogliomas, as well as secondary glioblastomas arising from these tumors (34). CIMP represents a distinct molecular phenotype in glioblastoma (35), that is associated with the proneural subgroup, where it shows extensive, coordinated hypermethylation at specific loci (35, 36). Notably, in their analysis, Northcott et al. found that *IDH1*-mutant SHH-MBs were determined to be CIMP+, highlighting the fact that these mutations play an epigenetic role comparable to those reported in other *IDH1*/*2*-mutant cancers (4). In line with this, we also observed a CpG island hypermethylation, further indicating a functional role of *IDH* mutation in reforming the DNA methylation pattern of MB. As a possible consequence of this reformed methylome, when analyzed with the DNA methylation classifier (13), the case only receives a relatively low classifier score (0.44), far below what is typically observed for SHH medulloblastoma (Figure 4).

*SMARCB1/INI1* encodes a chromatin remodeling protein (SNF5) that has been demonstrated to interact with various key proteins in several signaling pathways (37). SNF5 is identified as one of the key regulators of SHH signaling pathway through interacting with the glioma-associated oncogene family zinc finger-1 (GLI1), a crucial effector of SHH signaling pathway (38). Biallelic inactivation of *SMARCB1*/*INI1* results in aberrant expression or loss of SNF5 which is primarily seen in malignant rhabdoid tumors and ATRT (9) and recently reported in other tumor types such as epithelioid sarcoma, renal medullary carcinoma and schwannomatosis (37). Such *SMARCB1*/*INI1-*deficient tumors were found to show gene expression patterns associated with both activated SHH signaling pathway and GLI1 overexpression signatures, similar to SHH-activated MB (38).

Of notice, our case harbored a heterozygous missense somatic mutation (*SMARCB1*-R201G) that wasn't previously reported in any CNS tumor. This mutation is not predicted as inactivating mutation. Germline *SMARCB1* mutations are commonly associated with ATRT and renal rhabdoid tumors, in contrast to extra-renal rhabdoid tumors which typically harbor somatic biallelic deletions of *SMARCB1* (39). Interestingly, missense mutations of *SMARCB1*—whether germline or somatic—are virtually absent in all malignant rhabdoid tumors (39).

Sévenet et al. examined 299 different tumors for the prevalence of *SMARCB1* mutations (22). Among other brain tumors, they reported five out of 36 cases of MB with mutated *SMARCB1* gene (22). A contradictory study by Biegel et al. suggested that tumors harboring this mutation (especially in young age) are mostly ATRT misdiagnosed as MB (23). Indeed, two out of four MB/PNET samples with *SMARCB1* mutations were reclassified as ATRT after pathology revision, while other two samples (one MB and one PNET) had insufficient material for revision (23). Notably, three of the five cases reported by Sévenet et al. were less than 3 years old, and no pathology revision was performed in the study (22). In 2002, a dedicated study to settle this contradiction confirmed the absence of *SMARCB1* mutation in 90 MB samples (10).

Our case is diagnosed as a desmoplastic/nodular MB with characteristic SHH activation by virtue of detection of SHH targets (GAB1, p75NGFR) by immunohistochemistry. NGS identified a somatic missense *SMARCB1* mutation, yet there was no loss of SNF5 nuclear expression, so diagnosis of ATRT was ruled out.

## Conclusion

Our case shows that *IDH1* and missense *SMARCB1* mutations can be found concurrently in pediatric MB. While *IDH1* mutation represents the molecular basis of CIMP in gliomas, such role can't be confirmed in our MB case. Similarly, *SMARCB1* deletions / mutations result in loss of SNF5 expression and malignant rhabdoid tumor phenotype, yet in our case; nuclear expression of SNF5/INI1 was preserved. Whole exome and whole genome sequencing are becoming frequently used as clinical diagnostic tests at treatment institutions, and presumably they augment the accuracy and certainty of many diagnoses. However, the identification of rare mutations as an incidental finding will also increase, which might in-turn add to the diagnostic challenges in certain cases. The diagnostic significance as well as the clinical relevance of these mutations in the setup of histopathologically and genetically defined diagnosis need further investigation. Furthermore, such mutations need to be interpreted in the context of the different genetically defined entities of MB and larger studies are needed to explore what role these rare mutations might play in MB.

## Ethical statement

A written informed consent was obtained from the patient and his parents for the publication of this case report.

## Author contributions

AvB and ME-A design of the study. AvB, FG-P, and MA patient management with regards of diagnosis, treatment, and follow-up. AvB and ME-A collection and interpretation of patient data. ME-A and AvB wrote the first draft of the manuscript. TP, KE, DC, DS, DM, TM, ME-A, and AvB contributed to the histopathological diagnosis, methylation profiling, gene sequencing, and their interpretation of the presented case. Manuscript editing all authors. All authors read and approved the final manuscript.

### Conflict of interest statement

The authors declare that the research was conducted in the absence of any commercial or financial relationships that could be construed as a potential conflict of interest.

## Footnotes

^1^https://www.thermofisher.com/us/en/home/life-science/sequencing/next-generation-sequencing/ion-torrent-next-generation-sequencing-workflow/ion-torrent-next-generation-sequencing-select-targets/ampliseq-target-selection/ready-to-use-panels.html

## Appendix

**Table A1:** Sequencing results.

**Name of gene**	**Mutated allele frequency (%)**	**AA alteration**	**CDS^£^ mutation**	**Quality of coverage^∧∧^**	**Pathogenicity score^§^**
*IDH1*	46/24*	p.Arg132Cys	c.394C>T	A	5
*SMARCB1*	30	p.Arg201Gln	c.602G>A	A	4
*CDH11*	26	p.Ala625Thr	c.1873G>A	B	3
*EPHB4*	30	p.Asp119Asn	c.355G>A	A	1

* IDH1 mutation frequency reported twice, 46% with NGS over 50 genes, and 24% with NGS over 400 genes.

£ CDS: coding DNA sequence.

∧∧ Quality of coverage by NGS: The quality of coverage of a given gene is based on the number of reads, and their distribution along the amplicons, and is defined as follows:

Excellent: average number of reads ≥ 1,000 and Q95 ≥ 500.

Good: average number of reads ≥ 500 and Q95 ≥ 100.

Weak: average number of reads ≥ 100 and Q95 ≥ 100.

Inadequate: average number of reads < 100 or Q95 < 100.

The technical quality of detected variants is based on several criteria amongst others, the number and distribution of reads covering the variant and on the quality of sequence of the surrounding DNA; four categories are derived.

(A, excellent; B, good; C, weak; D, inadequate).

§ Pathogenicity score: 1, benign; 2, probably benign; 3, uncertain; 4, probably pathogenic; 5, pathogenic.
